# MNA-SF screening and GLIM diagnosis of malnutrition in geriatric patients – A cross-sectional multicenter study

**DOI:** 10.1016/j.jnha.2026.100878

**Published:** 2026-05-21

**Authors:** Stefanie Lemberger, Ilse Gehrke, Patrick Roigk, Wolfrid Schröer, Robert Speer, Hans-Jürgen Heppner, Katrin Singler, Peter Willschrei, Rainer Wirth, Dorothee Volkert

**Affiliations:** a Institute for Biomedicine of Aging, Friederich-Alexander-Universität Erlangen-Nürnberg, Nuremberg, Germany; b Clinic for Geriatrics and Geriatric Day Clinic, Bayreuth Hospital, Medical Campus Upper Franconia (MCO), Bayreuth, Germany; c Chair of Geriatrics, Friedrich-Alexander-Universität Erlangen-Nürnberg, Bayreuth, Germany; d Department of Geriatrics, Kliniken Sindelfingen, Klinikverbund Südwest, Sindelfingen, Germany; e Department of Geriatrics and Clinic for Geriatric Rehabilitation, Robert-Bosch-Krankenhaus GmbH, Stuttgart, Germany; f Sana Kliniken Duisburg GmbH, Duisburg, Germany; g Department of Medicine 2 - Geriatrics, Klinikum Nürnberg, Paracelsus Medical University, Nuremberg, Germany; h Department of Geriatric Medicine, Klinikum Fürth, Fürth, Germany; i Department of Geriatric Medicine, Evangelisches Krankenhaus Essen-Steele - KEM, Essen, Germany; j Department for Geriatric Medicine, Marien Hospital Herne, University Hospital Ruhr-University Bochum, Herne, Germany

**Keywords:** Malnutrition, GLIM, MNA-SF, Geriatric, Screening

## Abstract

**Objectives:**

To examine the relationship between the Mini Nutritional Assessment Short Form (MNA-SF) screening for malnutrition and the Global Leadership Initiative on Malnutrition (GLIM) diagnosis and the difference between the globally agreed GLIM two-step approach with prior screening (GLIMs+) and the alternative one-step approach without prior screening (GLIMs−) in a large group of geriatric patients.

**Design and setting:**

Cross-sectional multicenter study in eight geriatric institutions in Germany.

**Participants:**

681 geriatric patients.

**Measurements:**

Patient characteristics, MNA-SF and GLIM criteria were assessed in all patients using standardized questionnaires based on routine geriatric assessments. Malnutrition prevalence rates, Cohen’s kappa and diagnostic test parameters were calculated to compare MNA-SF results with GLIMs− and GLIMs− with GLIMs+.

**Results:**

88.1 % of all patients (83 ± 6.7 years; 68.0 % women) were MNA-SF positive (38.8 % malnourished, 49.3 % at risk). GLIMs− malnutrition was diagnosed in 44.6 %, GLIMs+ in 43.5 % of all patients. 8 patients (1.2 %) were normal according to MNA-SF and malnourished according to GLIMs− (false negative). Sensitivity of MNA-SF compared to GLIMs− was 0.97, specificity 0.19. Sensitivity of GLIMs− compared to GLIMs+ was 1.00, specificity 0.98. Cohen’s kappa indicated slight agreement between MNA-SF and GLIMs− (κ = 0.153) and near-perfect agreement between GLIMs− and GLIMs+ (κ = 0.976).

**Conclusion:**

The very good agreement between GLIMs− and GLIMs+ may argue in favor of direct GLIM diagnosis without prior screening, but prior MNA-SF screening saved the effort of GLIM testing in 12 % of all patients with only very few false negatives and also identifies the large group of at-risk patients who may benefit from preventive interventions.

## 1 Introduction

Malnutrition is highly prevalent among the geriatric population and results from a complex and multifactorial interplay between the physiological changes of aging, comorbidities and individual and environmental determinants, as well as other less well-understood factors [1]. It is strongly associated with increased risk of sarcopenia, morbidity and mortality, diminished quality of life, acute muscle wasting and prolonged or recurrent hospitalizations [[1], [2], [3]]. As a result, malnutrition not only has significant adverse consequences for individuals but also imposes a substantial economic burden on healthcare systems and societies [1,4].

Considering demographic changes, characterized by an increase in older individuals and thus geriatric patients [5], there is an urgent need to address malnutrition in geriatrics. Enhancing nutritional care for older people is crucial for preventing and treating malnutrition, promoting health and reducing the burden on social systems.

To achieve this, it is essential to identify individuals who could benefit from timely interventions. The Global Leadership Initiative on Malnutrition (GLIM) introduced a two-step diagnostic approach to standardize this process globally. The first step is to use a validated screening tool, such as the Mini Nutritional Assessment Short Form (MNA-SF), to detect patients at risk of malnutrition. The second step is to confirm or exclude the diagnosis of malnutrition in these patients based on three phenotypic and two etiological criteria [6]. The GLIM approach is well established and was recently overall confirmed [7]. It has been applied in older populations, and acceptable validity and good prognostic properties have been reported [8,9]. There is, however, still a need for clarification regarding the screening step [7]. Besides reported differences in prevalence rates when using different screening tools [10], discrepant results of applying the GLIM criteria in all patients or only in those identified as at risk of malnutrition or malnourished by prior screening were recently highlighted in a systematic review [11].

The necessity of screening as a first step is being questioned especially in geriatric patients, because of the very high prevalence of positive screening results [12], and several authors suggest omitting the screening step for high-risk populations [10,[13], [14], [15], [16]]. The question of whether to use GLIM with or without prior screening (two-step versus one-step approach) is particularly relevant in daily clinical practice to ensure optimal care for geriatric patients while efficiently utilizing staff capacity and time resources.

The aim of this study is to examine the relationship between MNA-SF screening and the GLIM diagnosis in a large group of geriatric patients. Specifically, the difference between the GLIM two-step approach with prior screening (GLIMs+) and the one-step approach without prior screening (GLIMs−) and thus the necessity of screening as a first step of the GLIM diagnosis is of interest. As malnutrition prevalence may vary in different geriatric health care settings [8,17], potential differences in the relation between MNA-SF and GLIM across different geriatric settings will be addressed.

## 2 Methods

### 2.1 Study design and participants

In this cross-sectional multicenter study in Germany, eight study centers (Herne, Essen, Duisburg, Stuttgart, Donaueschingen, Munich, Bayreuth, Nuremberg) participated with the aim of an approximate case number of 50 patients per center. Subjects were recruited in geriatric acute care hospitals (Herne, Essen, Duisburg, Donaueschingen, Munich, Bayreuth), in two geriatric day-hospitals (Nuremberg, Duisburg) and one mobile geriatric rehabilitation unit (Stuttgart). Consecutively admitted patients aged 65 or older were included. Exclusion criteria were terminal condition, inability to be weighed, amputation, severe fluid imbalance, dialysis and dependence on artificial nutrition for more than two weeks.

The first center (Nuremberg) started recruitment in May 2022, the last (Bayreuth) in February 2023. Data was collected by a medical doctor/student at each study center by using standardized questionnaires: MNA-SF and a custom-designed questionnaire for this study, including patient characteristics and GLIM malnutrition criteria. Data was taken from the patient record (routine examinations on admission) as far as available to minimize the burden for the patient and the examiner.

The study complies with the principles of the Declaration of Helsinki and received approval from the Ethics Committee of Friedrich-Alexander-Universität Erlangen-Nürnberg (22-144_1-B) from 09 February 2023. Written consent of the patient or the legal representative was obtained.

### 2.2 Patient characteristics

The patient’s age, gender and primary diagnosis were documented. Physical status was evaluated by the Clinical Frailty Scale with a maximum of 9 points indicating terminal illness [18] and by the Barthel index of basic activities of daily living with a score range from 0 to 100, where 100 points means complete independence [19]. Cognition was assessed by Mini-Mental State Examination (MMSE) [20], Montreal Cognitive Assessment (MOCA) [21] or Dementia Detection (DEM TECT) [22] depending on the clinical routine in the respective center and categorized into no (MMSE >23; MOCA >16; DEM TECT >8), moderate (MMSE 19–23; MOCA 10–16; DEM TECT 5–8) and severe (MMSE <19; MOCA <10; DEM TECT 0–4) impairment [23]. Depressive symptoms were examined by Depression in Old Age (DiA-S) [24] or Geriatric Depression Scale (GDS-15) [25] and interpreted as follows: Depression probable: DiA-S ≥4; GDS ≥6. The inflammatory marker C-reactive protein (CRP) was taken from routine laboratory tests, and levels of ≥30 mg/L were defined as indicative of inflammation [26].

### 2.3 Malnutrition screening and diagnosis

Malnutrition screening was performed by the MNA-SF questionnaire, comprising 6 questions about the decrease in food intake over the past three months due to loss of appetite, digestive issues, dysphagia or chewing difficulties as well as weight loss in the last 3 months, mobility, acute illness or stress, neuropsychological problems, specifically dementia and/or depression, and body mass index (BMI). A screening result is obtained by adding up the points for each question. The maximum score is 14 points. The patient shows a normal nutritional status in the range of 12–14 points. The risk of malnutrition is defined in the range of 8−11 points and a score below 8 points indicates malnutrition [27]. For all patients in geriatric acute care, the question MNA-SF D “Acute illness or psychological stress during the last three months” was answered affirmative, with a score of 0, due to the current acute illness event. Patients with malnutrition and patients at risk of malnutrition were considered MNA-SF positive.

GLIM criteria were assessed for every patient, regardless of the MNA-SF screening result, as follows [6]:

Phenotypic criteria: Body height and weight were measured to the nearest cm and 0.1 kg, respectively. BMI was calculated as body weight/body height² (kg/m^2^) and rated “low” for values below 20 kg/m^2^ in persons younger than 70 years and below 22 kg/m^2^ in persons aged 70 or older. Weight loss was asked in kg during the last 3, 6 or >6 months and percentage weight loss calculated. Weight loss was rated positive in case of >5 % within 6 months and >10 % beyond 6 months. Muscle mass was estimated by calf circumference (CC) and considered low if CC was <33 cm in male patients and <32 cm in female patients [7]. CC was measured in centimeters at the widest part of the right lower leg, with the knee bent at a 90-degree angle, using a flexible tape. If the measurement of the right leg was not feasible, the left leg was assessed and documented accordingly.

Etiological criteria: Food intake was classified as reduced if the rough estimate “<50 % of requirements for >1 week or any reduction for >2 weeks” was affirmed. Assimilation was considered positive in case of any chronic bowel disease that interferes with food intake, digestion and/or nutrient absorption. The inflammation criterion was determined by the patient’s clinical presentation based on medical examinations. Acute and chronic inflammation were documented separately and classified as either severe or mild, based on examples of diseases in each category, as in the original GLIM publication [6].

Malnutrition was diagnosed if at least one etiological criterion and one phenotypic criterion were present [6].

### 2.4 Data analysis

Participants’ data were pseudonymized in each center and sent to the principal investigator who merged all data and checked them for plausibility. Implausible values were clarified in consultation with the study centers and, if they could not be clarified, were changed to "missing". The data processing used the program Microsoft® Excel® for Microsoft 365 (Microsoft Corporation 2023, Version 2511) and the data analysis software IBM SPSS Statistics (Version 29.0.0.0) and complies with current data protection regulations (GDPR) [28].

Patients with missing data in the key variables MNA-SF or BMI were excluded. Missing values for cognition and depression are reported in a separate category. In cases of missing information for single GLIM criteria, normal values were assumed (weight loss n = 7, reduced muscle mass n = 17, reduced intake n = 10).

Patient characteristics, MNA-SF results and individual and combined GLIM criteria are reported as absolute and relative frequencies for categorical variables and as median value with interquartile range (IQR) for continuous variables, including age, Clinical Frailty Scale, CRP, BMI and Barthel index (all not normally distributed).

All results are presented for the total study group and separately by setting, for acute care, day hospital and mobile rehabilitation.

Group differences were examined by Pearson’s Chi-squared test for categorial variables and the non-parametric Kruskal–Wallis test for continuous variables. P values <0.05 were considered statistically significant. If significant, pairwise comparisons of the three subgroups were performed with Bonferroni correction (α = 0.0167) using Pearson’s Chi-squared test for categorical and Mann–Whitney *U* test for continuous variables.

Cross-tables were created to explore the relationship between MNA-SF results and GLIMs− diagnosis and between GLIMs− and GLIMs+. Agreement was assessed using Cohen’s kappa with 95 % confidence interval (CI) with combined MNA-SF positive groups and interpreted as follows: 0.01–0.20 = slight agreement; 0.21–0.40 = fair agreement; 0.41–0.60 = moderate agreement; 0.61–0.80 = substantial agreement; 0.81–0.99 = near-perfect agreement; and 1.00 = perfect agreement. The diagnostic test parameters sensitivity, specificity, positive predictive value (PPV), negative predictive value (NPV) and accuracy with 95 % CI of the MNA-SF in relation to the GLIMs− diagnosis as well as GLIMs− compared to the GLIMs+ diagnosis were calculated as follows [29]: Sensitivity = (true positive)/(true positive + false negative) Specificity = (true negative)/(false positive + true negative) Positive Predictive Value = true positive/(true positive + false positive) Negative Predictive Value = true negative/(false negative + true negative) Accuracy = (true positive + true negative)/all participants

## 3 Results

### 3.1 Patient characteristics

A total of 687 patients participated in the study. Six patients were excluded due to missing values of BMI or MNA-SF resulting in a final sample of 681 patients with a median age of 84 (IQR 79−88) years and a median BMI of 25.2 (22.2−29.3) kg/m^2^. About two thirds of the sample were women. 504 were acute care, 131 day-hospital and 46 mobile rehabilitation patients (Table 1). Depression was probable in approximately one-third with minimal differences between subgroups. Also, around one-third showed moderate to severe cognitive impairment. All patients were multimorbid. The most common primary diagnoses were fractures (24.4 %), predominantly hip fractures (11.2 %), gait instability including falls (12.6 %), cardiac disease (10.8 %, mainly decompensated heart failure (6.8 %)), and pain syndrome (9.4 %). About one quarter of the sample had elevated CRP levels. Day hospital patients had a higher BMI and Barthel index, lower frailty and CRP levels, and a lower rate of cognitive impairment compared to the other subgroups. Also, the day hospital patients were significantly younger than the mobile rehabilitation patients, their most common primary diagnoses were gait instability (36.6 %) and pain syndrome (19.8 %). Among patients in mobile rehabilitation, fractures were the most common primary diagnosis (41.3 %) followed by gait instability (17.4 %). In the acute care setting, fractures as well represented the most prevalent diagnosis (28.4 %), followed by cardiac diseases (13.3 %), thereby 9.1 % with decompensated heart failure.

**Table 1:** Patient characteristics [median (interquartile range) or % (*n*)].

	Total (*n* = 681)	Acute care (*n* = 504)	Day hospital (*n* = 131)	Mobile rehabilitation (*n* = 46)	P value*
Women	68.0 (463)	66.5 (335)	69.5 (91)	80.4 (37)	0.139
Age (years)	84 (79−88)	84 (80−88) ^a.b^	83 (79−86) ^a^	86 (80−90) ^b^	0.012
BMI (kg/m^2^)	25.2 (22.2−29.3)	24.8 (22.0−29.0)^a^	26.4 (23.7−30.0)^b^	23.9 (20.9−27.2)^a^	0.003
Cognitive impairment		^a^	^b^	^a^	<0.001
Severe	8.1 (55)	^a^ 8.5 (43)	^b^ 4.6 (6)	^a^ 13.0 (6)	
Moderate	24.4 (166)	27.2 (137)	16.0 (21)	17.4 (8)	
no impairment	59.5 (405)	55.8 (281)	79.4 (104)	43.5 (20)	
no information	8.1 (55)	8.5 (43)	0.0 (0)	26.1 (12)	
Depression					0.636
Probable	31.1 (212)	29.8 (150)	36.6 (48)	30.4 (14)	
Improbable	62.0 (422)	61.9 (312)	62.6 (82)	60.9 (28)	
no information	6.9 (47)	8.3 (42)	0.8 (1)	8.7 (4)	
Barthel index	55 (40−80)	50 (35−60) ^a^	90 (83−100) ^b^	50 (30−65) ^a^	<0.001
Clinical Frailty Scale	5 (4−6)	6 (5−6) ^a^	4 (4−5) ^b^	6 (5−7) ^a^	<0.001
C-reactive protein	11 (3−30)	17 (6−41) ^a^	5 (1−10) ^b^	0.1 (0.1−4) ^c^	<0.001
≥30 mg/l	26.3 (179)	33.8 (170)	2.3 (3)	13.0 (6)	

^a.b.c^ different superscripts indicate significant differences, identical superscripts indicate no difference.

* Pearson’s Chi^2^ or Kruskal–Wallis-Test; if p <0.05 pairwise comparisons by Pearson’s Chi^2^ or Mann–Whitney *U* test.

### 3.2 Prevalence of malnutrition and GLIM criteria

According to MNA-SF, 38.8 % of all patients were malnourished and 49.3 % at risk of malnutrition, together 88.1 % MNA-SF positive patients. In acute care 45.4 % were malnourished and 48.8 % at risk of malnutrition (94.2 % MNA-SF positive). In contrast, only 13.0 % of geriatric day hospital patients were malnourished as per MNA-SF, and 64.9 % MNA-SF positive. In the group of mobile rehabilitation patients 39.1 % were malnourished according to MNA-SF and 87.0 % MNA-SF positive (Table 2).

**Table 2:** Cross-tables of GLIMs− (without prior screening) and MNA-SF categories in geriatric patients [*n* (%)] with Cohen’s Kappa (95 % CI).*

	MNA-SF malnutrition (<8 p.)	MNA-SF at risk (8−11 p.)	MNA-SF normal (>11 p.)	Sum
A) Total study group (Kappa 0.153 [0.11–0.19])				
GLIMs− positive	189	107	8	304 (44.6%)
GLIMs− negative	75	229	73	377 (55.4%)
Sum	264 (38.8%)	336 (49.3%)	81 (11.9%)	681 (100.0%)

B) Acute care (Kappa 0.09 [0.05–0.13])				
GLIMs− positive	167	81	3	251 (49.8%)
GLIMs− negative	62	165	26	253 (50.2%)
Sum	229 (45.4%)	246 (48.8%)	29 (5.8%)	504 (100.0%)

C) Day hospital (Kappa 0.269 [0.14–0.39])				
GLIMs− positive	15	22	5	42 (32.1%)
GLIMs− negative	2	46	41	89 (67.9%)
Sum	17 (13.0%)	68 (51.9%)	46 (35.1%)	131 (100.0%)

D) Mobile rehabilitation (Kappa 0.09 [0.01–0.17])				
GLIMs− positive	7	4	0	11 (23.9%)
GLIMs− negative	11	18	6	35 (76.1%)
Sum	18 (39.1%)	22 (47.8%)	6 (13.0%)	46 (100.0%)

GLIMs− = Global Leadership Initiative on Malnutrition without prior screening, CI = confidence interval, MNA-SF = Mini Nutritional Assessment Short Form ®.

* MNA-SF malnutrition and at risk combined.

The GLIM diagnosis of malnutrition without prior screening (GLIMs−) was met in 44.6 % of the total study group, in 49.8 % acute care, 32.1 % day hospital and 23.9 % mobile rehabilitation patients. With prior screening (GLIMs+) the prevalence rates were 43.5 % in the total group and 49.2 %, 28.2 % and 23.9 % in the subgroups, respectively (Table 3).

**Table 3:** Prevalence of GLIM criteria and GLIM malnutrition in geriatric patients [% (*n*)].

	Total (*n* = 681)	Acute care (*n* = 504)	Day hospital (*n* = 131)	Mobile rehabilitation (*n* = 46)	P value*
**Phenotypic criteria**					
Weight loss **	25.6 (174)	27.6 (139) ^a^	23.7 (31) ^a.b^	8.7 (4) ^b^	0.017
Low BMI ***	22.8 (155)	24.8 (125) ^a^	12.2 (16) ^b^	30.4 (14) ^a^	0.004
Low muscle mass****	32.3 (220)	37.5 (189) ^a^	13.7 (18) ^b^	28.3 (13) ^b^	<0.001
**At least one phenotypic criterion**	**51.2 (349)**	**55.8 (281) ^a^**	**35.1 (46) ^b^**	**47.8 (22) ^a.b^**	<0.001
**Etiological criteria**					
Reduced food intake/assimilation	55.2 (376)	59.7 (301) ^a^	42.7 (56) ^b^	41.3 (19) ^b^	<0.001
Inflammation	65.9 (449)	68.1 (343) ^a^	73.3 (96) ^a^	21.7 (10) ^b^	<0.001
**At least one etiological criterion**	**82.7 (563)**	**84.9 (428) ^a^**	**85.5 (112) ^a^**	**50.0 (23) ^b^**	<0.001
**GLIMs− malnutrition**	**44.6 (304)**	**49.8 (251) ^a^**	**32.1 (42) ^b^**	**23.9 (11) ^b^**	<0.001
**GLIMs+ malnutrition**	**43.5 (296)**	**49.2 (248) ^a^**	**28.2 (37) ^b^**	**23.9 (11) ^b^**	<0.001

^a.b^ different superscripts indicate significance difference, identical superscripts indicate no difference.

GLIM = Global Leadership Initiative on Malnutrition, GLIMs− =GLIM malnutrition without prior MNA-SF screening (one-step approach), GLIMs+ = GLIM malnutrition with prior MNA-SF screening (two-step approach).

Lines in bold contain combined criteria.

* Pearsons Chi^2^ test.

** >5 % within 6 months, >10 % beyond 6 months.

*** Body mass index (BMI) <20 kg/m^2^ if age <70 years, <22 kg/m^2^ if age ≥70 years.

**** Calf circumference <33 cm for men, <32 cm for women.

At least one phenotypic GLIM criterion was observed in 51.2 % of the total sample, in day hospital patients significantly less often than in acute care patients (Table 3). All three individual phenotypic criteria were present in about a quarter to one third of the total sample. Of note here are the lower weight loss prevalence in mobile rehabilitation than in acute care patients and the less frequent reduction in BMI and CC in day hospital patients than in both other subgroups (Table 3).

At least one etiological GLIM criterion was present in 82.7 % of the total study group resulting from inflammation in about two thirds and reduced food intake or assimilation in more than half of the sample. Interestingly, only half of the mobile rehabilitation patients show at least one etiological criterion, mainly because of a much lower rate of inflammation. 60 % of acute care patients show reduced food intake/assimilation compared to only about 40 % in day hospital and mobile rehabilitation patients (Table 3).

### 3.3 Agreement between GLIM and MNA-SF

Cross-tables of GLIMs− and MNA-SF categories are presented in Table 2, diagnostic test performance indicators in Table 4, for the total study group and for the three subgroups, respectively.

**Table 4:** Diagnostic test parameters (with 95 % confidence intervals).

A) MNA-SF (malnutrition and at risk vs. normal) compared to GLIMs− malnutrition (without prior screening).					
	Sensitivity	Specificity	PPV	NPV	Accuracy
Total (*n* = 681)	0.97 (0.95−0.99)	0.19 (0.16−0.24)	0.49 (0.45−0.53)	0.90 (0.82−0.95)	0.54 (0.50−0.58)
Acute care (*n* = 504)	0.99 (0.97–1.00)	0.10 (0.07–0.15)	0.52 (0.48–0.57)	0.90 (0.74–0.96)	0.54 (0.50–0.59)
Day hospital (*n* = 131)	0.88 (0.75–0.95)	0.46 (0.36–0.56)	0.44 (0.33–0.54)	0.89 (0.77–0.95)	0.60 (0.51–0.68)
Mobile rehabilitation (*n* = 46)	1.00 (0.74–1.00)	0.17 (0.08–0.33)	0.28 (0.16–0.43)	1.00 (0.61–1.00)	0.37 (0.25–0.51)

B) GLIMs− malnutrition (without screening) compared to GLIMs+ malnutrition (with prior screening)					
	Sensitivity	Specificity	PPV	NPV	Accuracy
Total (*n* = 681)	1.00 (0.99−1.00)	0.98 (0.96−0.99)	0.97 (0.95−0.99)	1.00 (0.99−1.00)	0.99 (0.98−0.99)
Acute care (*n* = 504)	1.00 (0.98–1.00)	0.99 (0.97–1.00)	0.99 (0.97–1.00)	1.00 (0.99–1.00)	0.99 (0.98–1.00)
Day hospital (*n* = 131)	1.00 (0.91–1.00)	0.95 (0.88–0.98)	0.88 (0.75–0.95)	1.00 (0.96–1.00)	0.96 (0.91–0.98)
Mobile rehabilitation (*n* = 46)	1.00 (0.74–1.00)	1.00 (0.90–1.00)	1.00 (0.74–1.00)	1.00 (0.90–1.00)	1.00 (0.92–1.00)

GLIM = Global Leadership Initiative on Malnutrition, GLIMs− Global Leadership Initiative on Malnutrition without screening, GLIMs+ Global Leadership Initiative on Malnutrition with prior screening, MNA-SF = Mini Nutritional Assessment Short Form ®, NPV = negative predictive value, PPV = positive predictive value.

In the total study group, eight patients (1.2 %) were classified as normal according to MNA-SF but as malnourished according to GLIMs− (false negative), with no mobile rehabilitation patients being incorrectly classified as non-malnourished. 71.6 % of those classified as MNA-SF malnourished (189/264) and 31.8 % of those at risk (107/336) were diagnosed with malnutrition by GLIM, together nearly half (296/600 = 49.3 %) of the MNA-SF positive patients (Table 2). Cohen’s kappa indicated slight agreement between MNA-SF and GLIMs− in the total group (κ = 0.153), fair agreement in day hospital patients (κ = 0.269) and very low agreement in mobile rehabilitation and acute care (both κ = 0.09).

Sensitivity of the MNA-SF for identifying GLIM-defined malnutrition was high across all settings, reaching 0.97 in the total sample, 0.99 in acute care, and 1.00 in mobile rehabilitation patients (Table 4-A). Similarly, the NPV was consistently high: close to or above 0.90 in the overall cohort and all settings (Table 4-A). In contrast, specificity was low overall (0.19), with even lower values observed in acute care and mobile rehabilitation, whereas higher specificity was found in day hospital patients (0.46), albeit at the expense of sensitivity (0.88). PPV and accuracy ranged from 0.44 to 0.60 in the overall sample, in acute care and in day hospital. However, both values were lower in mobile rehabilitation patients (PPV 0.28; accuracy 0.37).

The comparison of GLIMs− with GLIMs+ is shown in Table 5. Due to the two-step approach and the successive results, no patient can be GLIMs− negative but GLIMs+ positive. Cohen’s kappa indicated near-perfect agreement in the total group, in acute care and day hospital patients and perfect agreement in mobile rehabilitation. All diagnostic test parameters were close to one in the total sample and in all settings (Table 4-B).

**Table 5:** Cross-tables of GLIMs− (without prior screening) and GLIMs+ (with prior screening) in geriatric patients [*n* (%)] with Cohen’s Kappa (95 % CI).

A) Total study group (Kappa 0.976 [0.96–0.99])			
	GLIMs− positive	GLIMs− negative	Sum
GLIMs+ positive	296	0	296 (43.5%)
GLIMs+ negative	8	377	385 (56.5%)
Sum	304 (44.6%)	377 (55.4%)	681 (100.0%)

B) Acute care (Kappa 0.988 [0.97–1.00])			
	GLIMs− positive	GLIMs− negative	Sum
GLIMs+ positive	248	0	248 (49.2%)
GLIMs+ negative	3	253	256 (50.8%)
Sum	251 (49.8%)	253 (50.2%)	504 (100.0%)

B) Day hospital (Kappa 0.91 [0.83–0.98])			
	GLIMs− positive	GLIMs− negative	Sum
GLIMs+ positive	37	0	37 (28.2%)
GLIMs+ negative	5	89	94 (71.8%)
Sum	42 (32.1%)	89 (67.9%)	131 (100.0%)

B) Mobile rehabilitation (Kappa 1.00 [1.00–1.00])			
	GLIMs− positive	GLIMs− negative	Sum
GLIMs+ positive	11	0	11 (23.9%)
GLIMs+ negative	0	35	35 (76.1%)
Sum	11 (23.9%)	35 (76.1%)	46 (100.0%)

GLIMs− = Global Leadership Initiative on Malnutrition without prior screening, GLIMs+ = Global Leadership Initiative on Malnutrition with prior screening, CI = confidence interval.

## 4 Discussion

In this large sample of geriatric patients from three healthcare settings, a malnutrition prevalence of 45 % was observed using GLIM criteria without prior screening. Following prior malnutrition screening by MNA-SF, the prevalence was only 1 % lower (Table 3). All diagnostic test indicators and Cohen’s kappa also indicated a very high agreement between GLIM with and without prior screening (Table 4, Table 5). Further, the sensitivity and NPV of the MNA-SF to identify GLIM malnutrition was very high, in contrast, specificity in this regard was quite low at only about 0.2, and PPV as well as accuracy only about 0.5.

### 4.1 Prevalence of malnutrition

For screening we selected the MNA-SF which is the most commonly used tool and well established for geriatric patients [15]. MNA-SF was positive in 88 % of the patients, 39 % were malnourished and 49 % at risk of malnutrition, which is in very good agreement with a previous pooled data set analysis [17] and also in line with more recent results of 100 inpatients from two geriatric wards in Sweden, where 87 % were MNA-SF positive and 32 % MNA-SF malnourished [13]. Overall, the prevalence is in the upper range of other MNA-SF positive rates reported in connection with GLIM results in geriatric patients [[30], [31], [32], [33]].

The observed GLIM malnutrition prevalence rate is in the middle of the range of previously reported rates in geriatric patients. Prospectively assessed rates without prior screening and considering all GLIM criteria vary between 32 % and 58 % [10,12,13,[32], [33], [34], [35], [36], [37], [38]] with higher rates in onco-geriatric patients [32] and older patients admitted to an emergency department [33] and lower rates in geriatric outpatients [34].

Noteworthy in our study is the only 1 % lower rate when following the two-step approach. A larger decrease from 58 % to 51 % (also based on MNA-SF screening) was reported in geriatric acute care patients in Sweden [13]. In some studies, reporting only GLIM malnutrition following MNA-SF screening, GLIM prevalence rates of 50.3 % or less are reported [16,30,31,33,39]. Besides different sample characteristics, differences may also be due to different operationalization of the GLIM criteria [12,36].

### 4.2 GLIM criteria

In our sample, at least one phenotypic GLIM criterion was observed in nearly half of the patients, each single criterion in about one fourth to one third. In most patients (83 %) at least one of the two etiological criteria was present, inflammation/disease burden in about two thirds, reduced intake/assimilation in about half (Table 3).

The prevalence of reduced food intake/assimilation is in line with previous reports [13,40]. The high presence of inflammation can be attributed to acute diseases and multiple comorbidities in geriatric patients. Lower rates were reported based on laboratory parameters (e.g., CRP, albumin) [36,40]. However, using both approaches, Mateo Silleras et al. [36] found that lab-based prevalence rates exceeded those based on clinical assessment.

In our study, both etiological criteria, inflammation and reduced food intake/assimilation, were based on clinical evaluation, which is a pragmatic and practical approach that can be applied in everyday clinical practice and is regarded as a good option also by the GLIM committee [6,7,41]. However, a certain degree of subjectivity cannot be excluded, and more research is required to examine the agreement between clinical and more objective assessments.

Muscle mass was estimated by CC, also because of its simple measurement without technical tools and expertise for advanced body composition measures [41]. Due to its low burden for the patient, it is particularly suitable for geriatric patients [42]. However, cut-off values are still under discussion [6,7]. We used the cut-off values suggested by the GLIM consortium independent of age. Due to the decreasing muscle mass with age, a lower cut-off value might be justified for old people. Applying 31 cm as cut-off, which is sometimes used for geriatric patients [43], would result in a lower prevalence of reduced muscle mass and consequently in a lower malnutrition prevalence. On the other hand, the prevalences may be somewhat underestimated, as 17 patients (2.5 %) with missing values were assumed to have normal CC. A further underestimation of the prevalence of low muscle mass is likely due to the widespread occurrence of edema in geriatric patients, which may increase CC substantially. Ishida et al. [44] reported an increase in CC of 1.6 cm in older male and of 3.0 cm in older female patients with lower extremity edema compared to matched controls without edema. In our sample 25.0 % mild and 14.0 % significant edema were observed (12 values were missing). Reliable approaches to take these aspects into account in the future need to be developed.

### 4.3 Differences between geriatric settings

Overall, our results are determined by the patients from acute care departments, who represent almost three-quarters of the sample. With 94 % MNA-SF positive, 45 % MNA-SF malnutrition, 50 % GLIMs− and 49 % GLIMs+ malnutrition, prevalence rates are highest in this subgroup.

Mobile rehabilitation patients are unfortunately underrepresented, with only 46 patients, 7 % of the sample. Their results must therefore be interpreted with caution and cannot be considered representative. Interestingly, patient characteristics (Table 1) and prevalence of some GLIM criteria (Table 2) are similar to the acute care patients. MNA-SF results (87 % MNA-SF positive) are also remarkably similar to the acute care patients and in line with previous reports [17]. In contrast, GLIM-defined malnutrition was markedly less common (24 % with and without prior screening; Table 3) than in acute care patients and compared to other samples of geriatric rehabilitation patients [37,38]. This lower GLIM rate is due to a markedly lower prevalence of inflammation (and thus etiological GLIM criteria), which might be explained by the fact that some of the patients have already at least partially recovered from an acute event.

Day hospital patients represent 19 % of the total sample, they were younger and had less cognitive and physical impairment than acute care and mobile rehabilitation patients (Table 1), and with 65 % the lowest MNA-SF positive rate (Table 2), which explains the relatively high specificity of 0.46 (Table 4). The GLIM malnutrition prevalence of 32 % without and 28 % with prior MNA-SF screening is lower than in acute care patients and in line with other results from older outpatients [34]. In our sample, the lower prevalence is explained by less common phenotypic criteria of low BMI and reduced muscle mass and may be due to an overall better health and functional status of this subpopulation.

### 4.4 Screening followed by GLIM or “GLIM only”

According to our results, the proposed omission of the screening-step in the diagnosis of malnutrition (due to the high risk of malnutrition in geriatric populations) [10,[13], [14], [15], [16]] would result in a considerable proportion of patients at least at risk of malnutrition per MNA-SF being missed (false positive). Thus, the two-step approach is valuable in identifying these individuals who may benefit from preventive measures to eliminate existing risk factors, improve their nutritional situation and avoid the development of malnutrition.

The two-step approach offers resource savings because the more time-intensive GLIM assessment is not required for all patients, whereas screening is performed in all. On the other hand, the one-step approach would also save resources by eliminating the screening step but requires GLIM assessment for all patients.

The MNA-SF normal rate represents the proportion of patients who do not require further GLIM assessment, thereby optimizing time and resource allocation. This raises the question at what MNA-SF normal rate the two-step approach becomes worthwhile. Assuming that this is the case when the MNA-SF normal rate exceeds 10 %, this approach would be particularly beneficial in settings such as day hospitals (35.5 % MNA-SF normal) and also in mobile rehabilitation (13 % MNA-SF normal), while its relevance for acute care patients (6 % MNA-SF normal) is questionable. Consequently the “GLIM only” approach would be most suitable for acute geriatrics due to the high MNA-SF positive prevalence.

False negatives refer to patients who, despite being malnourished according to GLIM criteria, are not identified through the screening process and therefore do not receive treatment when following the two-step approach. Ideally, this rate should be as low as possible. The literature reports 3 % in 100 geriatric acute care patients, mean age 82 years [13] and 2 % in 490 patients aged 70 ± 16 years [35]. In our study, 1.2 % of all cases were false negative. Only three acute care patients (0.6 % of acute care) and five day-hospital patients (3.8 % of day hospital) would be incorrectly missed for a malnutrition diagnosis using the two-step approach, resulting in no treatment. This represents a negligible small patient group, where the lack of diagnosis and treatment seems acceptable, justifying the two-step approach.

The “false positive” rate consists of patients who screen positive (MNA-SF <11 points) but do not meet GLIM malnutrition criteria. These individuals have an impaired nutritional status according to MNA-SF and might benefit from early preventive interventions. In all three subgroups, this rate exceeds 10 %. 11 % of the whole sample (28 % of MNA-SF malnourished patients) are even malnourished according to MNA-SF but GLIM negative. In these patients, a more detailed analysis of the individual MNA-SF questions would be necessary and based on this, consideration of possible interventions to improve nutritional status or eliminate the risk.

The high prevalence of 88.1 % MNA-SF positivity, and a high number of true positives, as well as very low false negatives, confirm the finding of a high sensitivity of 0.97. The specificity of the screening tool is notably low. Consequently, healthy patients are frequently incorrectly identified as malnourished, a consequence of the substantial false positive rate of 44.6 %. This is primarily attributable to the false positives of the MNA-SF at risk group, which constitutes 33.6 % of the total study group. These individuals are classified as MNA-SF at risk positive, yet not yet malnourished, according to the GLIM criteria. The elevated rate of false positives also leads to a low PPV and an accuracy of 0.54. Due to the very few false negatives, the overall group has a high NPV of 0.90.

PPV was highest in acute care (0.52) alongside a NPV of 0.90, reflecting the highest malnutrition prevalence in this setting, as both predictive values are prevalence dependent. Accordingly mobile rehabilitation with the lowest prevalence exhibited a low PPV but a perfect NPV of 1.00. Accuracy was lowest in the mobile rehabilitation group, due to a high number of false positives. These findings can be attributed to the high MNA-SF positive prevalence, almost as high as in acute care patients in comparison to a low GLIM prevalence. MNA-SF is less influenced by inflammation than GLIM. Since mobile rehabilitation patients exhibit low inflammatory activity, GLIM scores are low, while MNA-SF results are comparatively higher. In comparison, the day hospital subgroup achieved the highest accuracy, corresponding to the fewest misclassifications.

Due to the fact that the MNA-SF questions on mobility and neuropsychological problems are part of the usual geriatric assessment, and the remaining MNA-SF questions largely overlap with the GLIM criteria, the two-step procedure with MNA-SF for all patients does not involve any significant additional effort and seems to be a justifiable and acceptable approach.

### 4.5 Strengths and limitations

In contrast to earlier research [8], in our study all GLIM criteria were assessed prospectively for every patient. GLIM criteria were defined pragmatically to allow easy assessment in everyday clinical practice. However, this could also have led to inaccuracies that need to be investigated more thoroughly in the future.

This study involves eight centers across various geriatric settings. As a result, we obtained a large, but heterogenous sample. This heterogeneity was addressed by setting-specific analyses. Data from geriatric day hospitals and mobile rehabilitation are considered a beginning to close a knowledge gap, as currently no literature is known comparing MNA-SF and GLIM in these geriatric settings. The relatively small sample size of 131 day-hospital and 46 mobile rehabilitation patients limit generalizability of the findings and requires future investigation of larger samples from these specific geriatric settings.

## 5 Conclusions

We found a very high sensitivity but low specificity of MNA-SF to identify malnutrition in a heterogenous sample of geriatric patients and near-perfect agreement with only 1 % difference between the GLIM malnutrition with and without prior screening. Prior screening saves the effort of GLIM diagnosis in 12 % of patients and results in only very few malnourished geriatric patients who are overlooked (1.2 % false negative**).** Furthermore, screening identifies a large group of patients at risk of malnutrition who may benefit from preventive measures (44.6 % MNA-SF positive and GLIM negative; 33.6 % MNA-SF at risk and GLIM negative). Therefore, we recommend to maintain the two-step approach for geriatric patients. The observed differences between the settings need to be substantiated by further studies.

## CRediT authorship contribution statement

**Stefanie Lemberger:** Data Curation, Formal analysis, Writing - Original Draft, Review & Editing. **Ilse Gehrke:** Data Curation. **Patrick Roigk:** Data Curation. **Wolfrid Schröer:** Data Curation. **Robert Speer:** Data Curation. **Hans-Jürgen Heppner:** Data Curation, Supervision. **Katrin Singler:** Data Curation. **Peter Willschrei:** Data Curation. **Rainer Wirth:** Conceptualization, Data curation. **Dorothee Volkert:** Conceptualization, Project administration, Supervision, Writing – Review & Editing.

All authors were involved in designing the study, critically read the manuscript and approved the final version.

## Declaration of generative AI and AI-assisted technologies in the writing process

During the preparation of this work the authors used Chat GPT (OpenAI) in order to improve language and readability. After using this service, the authors reviewed and edited the content as needed and take full responsibility for the content of the published article.

## Funding

The present research project received no specific funding.

## Data availability

Original data are available on reasonable request.

## Declaration of competing interest

RW received lecture-honoraria from Nutricia Danone. RS is employed by Danone Deutschland GmbH. The other authors declared to have no conflict of interest.
